# Lock and chop: A novel method for the generation of a PICK1 PDZ domain and piperidine‐based inhibitor co‐crystal structure

**DOI:** 10.1002/pro.3361

**Published:** 2018-01-30

**Authors:** Douglas J. Marcotte, Jean‐Christophe Hus, Charles C. Banos, Craig Wildes, Robert Arduini, Chris Bergeron, Catherine A. Hession, Darren P. Baker, Edward Lin, Kevin M. Guckian, Anthone W. Dunah, Laura F. Silvian

**Affiliations:** ^1^ Biotherapeutics and Medicinal Sciences Biogen Inc Cambridge Massachusetts; ^2^ Neurodegeneration and Repair Biogen Inc Cambridge Massachusetts; ^3^ Present address: Department of Brain and Cognitive Sciences Massachusetts Institute of Technology Cambridge Massachusetts 02139; ^4^ Present address: Sanofi Cambridge MA 02142

**Keywords:** class II PDZ binding motif, PICK1 PDZ domain crystal structure, PDZ domain small molecule inhibitors, proteolysis, PDZ domain inhibitor cocrystallization

## Abstract

The membrane protein interacting with kinase C1 (PICK1) plays a trafficking role in the internalization of neuron receptors such as the amino‐3‐hydroxyl‐5‐methyl‐4‐isoxazole‐propionate (AMPA) receptor. Reduction of surface AMPA type receptors on neurons reduces synaptic communication leading to cognitive impairment in progressive neurodegenerative diseases such as Alzheimer disease. The internalization of AMPA receptors is mediated by the PDZ domain of PICK1 which binds to the GluA2 subunit of AMPA receptors and targets the receptor for internalization through endocytosis, reducing synaptic communication. We planned to block the PICK1‐GluA2 protein–protein interaction with a small molecule inhibitor to stabilize surface AMPA receptors as a therapeutic possibility for neurodegenerative diseases. Using a fluorescence polarization assay, we identified compound BIO124 as a modest inhibitor of the PICK1‐GluA2 interaction. We further tried to improve the binding affinity of BIO124 using structure‐aided drug design but were unsuccessful in producing a co‐crystal structure using previously reported crystallography methods for PICK1. Here, we present a novel method through which we generated a co‐crystal structure of the PDZ domain of PICK1 bound to BIO124.

## Abstract 2

Interactive Figure 2, Interactive Figure 4 | PDB Code(s): 6BJN; 6BJO

## Introduction

Protein interacting with kinase C1 (PICK1) is a membrane protein composed of a N‐terminal PDZ domain and a C‐terminal BAR domain, and is expressed highly in the brain and peripheral tissues.^1^, ^2^ It functions as an intracellular trafficking protein involved in the internalization of amino‐3‐hydroxyl‐5‐methyl‐4‐isoxazole‐propionate (AMPA) type receptors which are localized on the surface of neurons and mediate synaptic communication.^3^, ^4^ Down regulation of surface AMPA receptors on neurons reduces synaptic communication leading to cognitive impairment in progressive neurodegenerative diseases such as Alzheimer disease.^5^ GluA2 is a subunit of AMPA type receptors and is composed of an extracellular N‐terminal ligand binding domain, a transmembrane domain, and an intracellular C‐terminal domain. The PDZ domain of PICK1 recognizes and binds to the intracellular PDZ binding motif on the c‐terminus of GluA2. The PICK1‐GluA2 protein–protein interaction leads to the trafficking of AMPA type receptor away from the neuron surface through endocytosis, thereby reducing synaptic communication.^6^, ^7^ We proposed to develop an inhibitor targeting the PICK1 PDZ domain‐GluA2 interaction using structure‐aided drug design for the treatment of neurodegenerative diseases through the stabilization of surface AMPA type receptors on neurons. PDZ domains provide a somewhat selective recognition function that serves to assemble protein complexes by binding PDZ binding motifs on target proteins. PDZ binding motifs are short, generally four amino acid stretches at the c‐terminus of a target protein. The nomenclature for PDZ peptide binding motifs classifies the last residue of the c‐terminus as the 0‐position and the residues n‐terminal to the 0‐position as negative positions.

Specificity between PDZ domains and the PDZ binding motifs are imparted by the −2 position of the motif, and the nature of the residue at this position is what classifies PDZ binding motifs into class I, class II, or class III motifs.^8^, ^9^ Class I sequence motifs have **S/T**‐X‐Φ‐COOH; class II sequence motifs have **Φ** ‐X‐ Φ ‐COOH, and class III sequence motifs have **D/E**‐X‐ Φ‐COOH; where Φ is a hydrophobic residue and X is any amino acid. This selectivity for PDZ binding motif classes has been attributed to the interaction between the −2 position of the PDZ binding motif with the first amino acid side chain on the αB helix (αB1) of the PDZ domain.^10^

**Figure 1:**
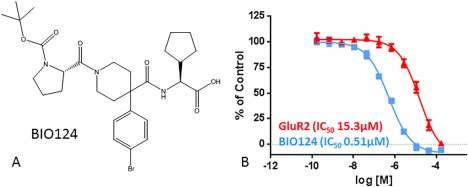
(A) 2D structure of BIO124. (B) IC50 curves for the GluR2 peptide and BIO124 for PICK1 as determined by the fluorescent polarization assay.

PICK1's PDZ domain is unique in its ability to bind both class I and II motifs, however, it binds class II motifs (i.e., GluA2 (S**V**KI)) with a 10‐fold‐higher affinity over class I motifs (i.e., PKCα (QSAV)).^9^, ^10^, ^11^ Our goal was to disrupt the protein–protein interaction between PICK1 and the higher affinity class II GluA2 PDZ binding motif. We used a fluorescence polarization assay for our high throughput screen (HTS) efforts to identify small molecule inhibitors that displaced a labeled GluA2 peptide from PICK1. We identified a novel class of piperidine‐based inhibitors that led us to BIO124 [Fig. 1(A)] a sub‐μM molar inhibitor of the PICK1‐GluA2 interaction.

In this study, we wished to understand the binding mode of the PICK1‐GluA2 inhibitor BIO124 by crystallography and NMR. Since the apo‐PICK1 PDZ domain was sparingly soluble, we were unable to form a complex with BIO124 suitable for structural studies. Here, we report a novel “lock and chop” method for producing a co‐crystal structure of PICK1's PDZ domain with the small molecule inhibitor BIO124 which enabled our understanding of its binding mode.

## Results and Discussion

### Developing a method to co‐crystallize PICK1 PDZ domain with BIO124

To enable a structure‐aided drug design approach to improve the potency of BIO124, we wished to understand the interaction of the inhibitor to the PDZ domain of PICK1 using x‐ray crystallography. We designed the PDZ domain of PICK1 for crystallization using the self‐binding extension method described previously for PICK1 and other PDZ domains.^18^ We fused the class 1 PDZ binding motif QSAV from PKCα to the C‐termini of the PICK1 PDZ domain to enhance solubility and aid crystallization. The purified PICK1 PDZ‐QSAV protein eluted as a dimer on size exclusion chromatography under reducing conditions indicating that we successfully designed an intramolecular interaction between the PDZ domains using the fused QSAV extension. Since the construct contained two reactive cysteines, we confirmed that the dimer could be “locked” by disulfide bridging when oxidized.^10^ The reaction was ∼90% complete after addition of H_2_O_2_ as assessed by nonreducing SDS‐PAGE and comparing the intensity of the monomer to dimer bands. The disulfide‐bridged PICK1 PDZ‐QSAV protein could be concentrated to 10 mg/mL and produced diffraction quality crystals in 2 days. This “locked” version of the PICK1 PDZ‐QSAV dimer crystallized much more readily than the unlocked reduced dimer. We hypothesize that the oxidized dimer is more stable to aggregation as seen from its increased thermal stability compared to the unlocked reduced dimer (Supporting Information Fig. S1).

Once we established that we could produce a crystallization‐competent PICK1 PDZ‐QSAV dimer, this protein was used for co‐crystallization with BIO124. The structure revealed electron density that confirmed that the dimer was locked by two disulfide bounds between C44(A)‐C46(B) and C44(B)‐C46(A) between the two PDZ domain chains of PICK1 (Supporting Information Fig. S2). However, because electron density for the fused QSAV tail from the dimeric partner peptide was visible in the peptide groove, this method failed in allowing BIO124 to displace the QSAV tail within the crystal lattice.

### Comparison of class I (QSAV) and class II (SVKI) peptide binding to PICK1

The crystal structure that was obtained in the attempt to co‐crystallize with BIO124 represents the first PICK1 structure bound to a class I PDZ binding motif (QSAV from PKCα). We wished to better understand the differences in the 10‐fold higher binding affinities for class II vs. class I motifs to PICK1 by comparing our PICK1 PDZ domain class I motif structure with structures of PICK1 PDZ domain bound to class II PDZ motifs from the PDB.^10^, ^18^, ^19^, ^20^

**Figure 2:**
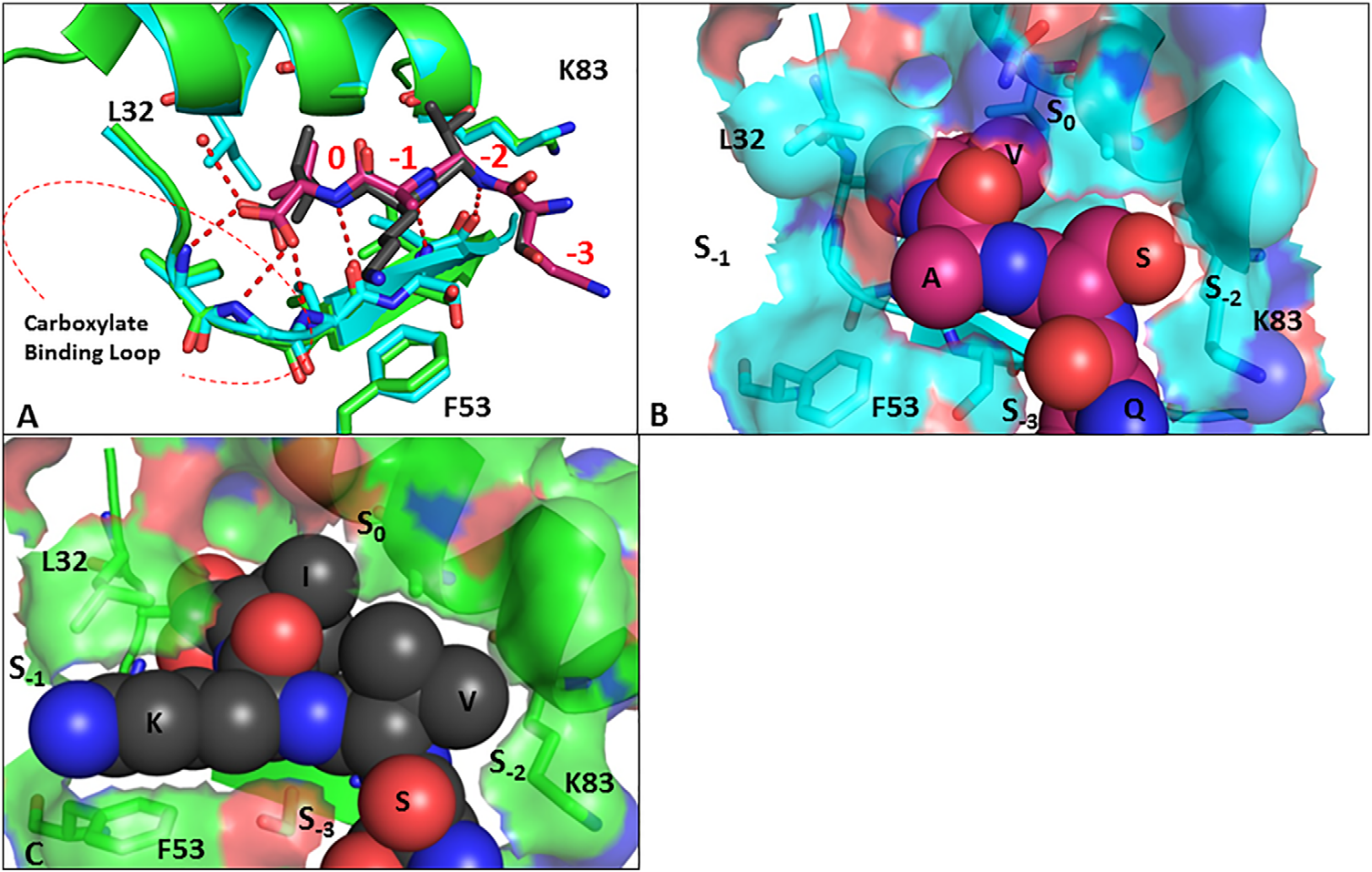
(A) Comparison of binding modes of Class I PKCα (QSAV (magenta)) peptide with Class II GluA2 (SVKI (black (PBD:3HPK))) with PICK1. Space filling representation of the PKCα (B) and GluA2 (C) peptides on the surface of PICK1. An interactive view is available in the electronic version of the article

The superposition of class II peptide bound PICK1 PDZ‐3C‐VYGIESVKI crystal structure (PDB code 3HPK) with our class I peptide bound PICK1 PDZ‐QSAV demonstrated few differences in the sidechain positions of the residues surrounding the peptide binding groove [Fig. 2(A)]. However, there were two hydrophobic “hotspots” that differ between the two peptides and could explain the differences in binding affinities [Fig. 2(B,C)]. The lysine on the GluA2 class II SVKI peptide buries into the S_–1_ sub‐pocket where it is involved in lipophilic interactions with Leu32 and Phe53. The class 1 PKCα QSAV peptide has an alanine at the −1 position and is unable to make this interaction. The second difference is between the polar and nonpolar sidechains at the −2 position. The polar serine of the class I QSAV does not make an interaction to K83 in the αB1 position in PICK1. In the class II SVKI, the hydrophobic valine sidechain at −2 position forms a lipophilic interaction with the aliphatic segment of the Lys83 sidechain in the S_–2_ subpocket. These two additional lipophilic interactions support the observations that class II peptides demonstrate higher binding affinities for PICK1 over class I peptides.

### BIO124 competes with the fused class 1 QSAV for binding in the peptide binding grove of PICK1 in solution

**Figure 3:**
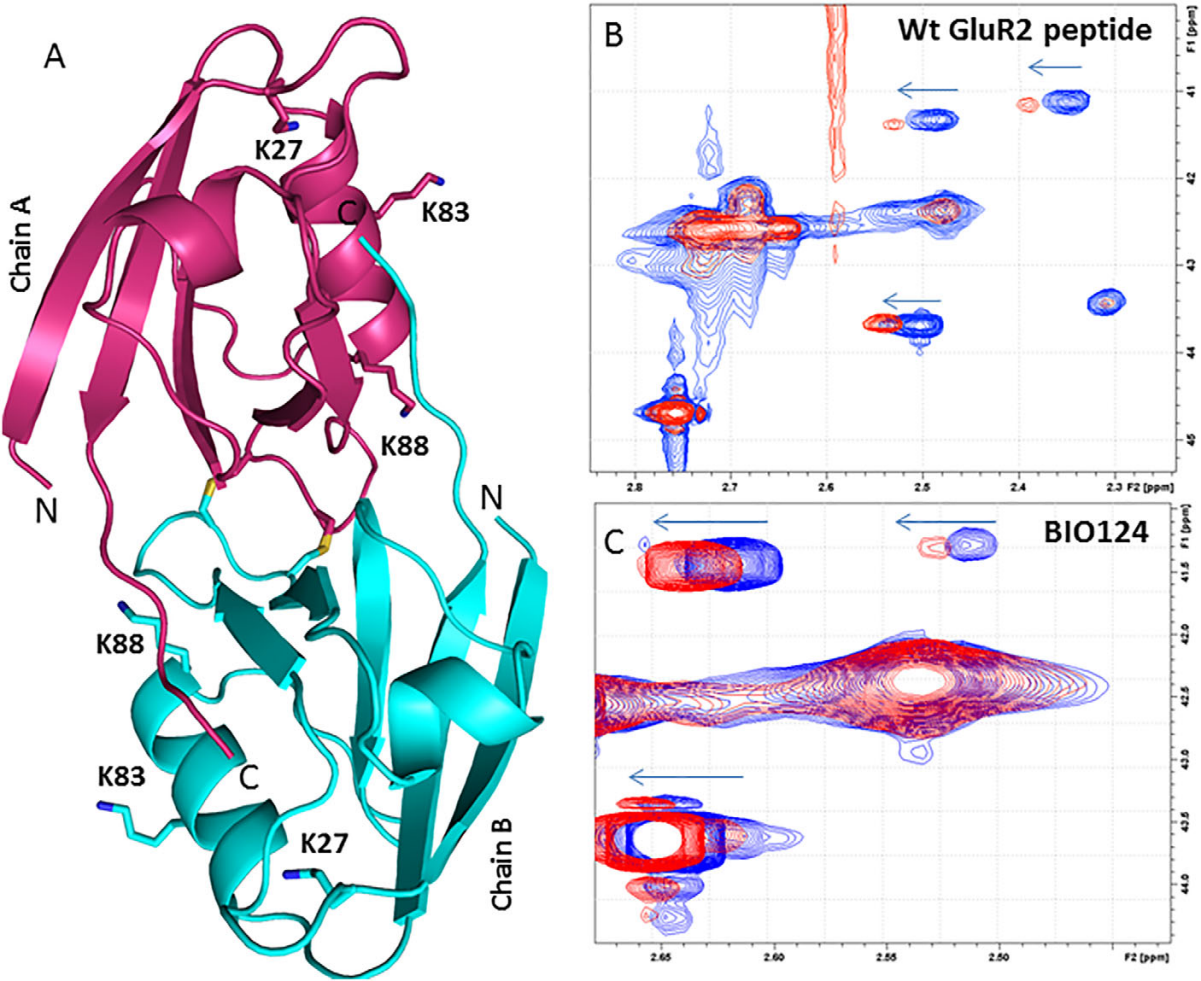
(A) Overall Structure of PICK1 PDZ‐QSAV with Lysines 6Å away from peptide binding pocket labeled. Shifts of methylated Lysines in 2D Protein NMR for (B) Wt GluR2 peptide and (C) BIO124.

To determine if BIO124 could reasonably compete with the neighboring QSAV tail in solution, we used two solution‐based methods to confirm that the compound was likely binding in the peptide binding groove for this construct. The first method used a protein‐based 2D NMR approach to confirm that the compound can compete out the QSAV for the peptide binding pocket. Lysine methylation was used to probe the effects of compound binding. Seven lysines are available for methylation in the PICK1 PDZ domain, of which three are near (less than 6Å) the binding pocket [Fig. 3(A)]. Upon binding to the GluA2 peptide, three peaks in the 19C‐HSQC showed chemical shift changes [Fig. 3(B)] which we reasoned to be the three closest lysines (K27, K83, and K88 to the peptide binding pocket of PICK1 [Fig. 3(A)]). Upon addition of BIO124 the same lysines' exhibit chemical‐shift changes [Fig. 3(C)]. The peaks that shift upon titration of the GluA2 peptide and BIO124 are the same, suggesting that the GluA2 peptide and BIO124 bind to the same peptide binding groove, confirming that this construct is capable of binding the compound and displacing the peptide extension in solution.

The second method that we used to confirm displacement of the neighboring QSAV peptide from the peptide binding pocket of PICK1 was through partial proteolysis. BIO124 was added at a 4.5‐fold molar excess to the “locked” PICK1 PDZ‐QSAV dimer to make the peptide extension accessible to bovine carboxypeptidase A (bCPA). Mass spectrometry showed that a 16‐hour digestion with catalytic amounts of bCPA resulted into >95% removal of the 0 and −1 position residues of the QSAV tail [Supporting Information Fig. S3(A,B)]. In contrast, in the same experiment in the absence of the compound, no proteolysis took place [Supporting Information Fig. S3(C)]. This suggests that the compound is in fact displacing the peptide in solution, thereby making it accessible to bCPA cleavage.

We hypothesized that if we could exploit the proteolysis method to irreversibly prevent the QSAV peptide from rebinding to the carboxylate binding loop, we could shift the binding equilibrium from the peptide bound form to the BIO124 bound form for crystallization trials. We hypothesized that if we used PICK1 PDZ‐QSAV crystals as seeds, we could grow diffraction‐ quality crystals of the proteolyzed PICK1‐PDZ‐QS BIO124 complex. Interestingly, if the dimer was not locked by oxidation, no crystals were observed, suggesting that the dimer packing needed to be maintained to obtain quality crystals.

**Figure 4:**
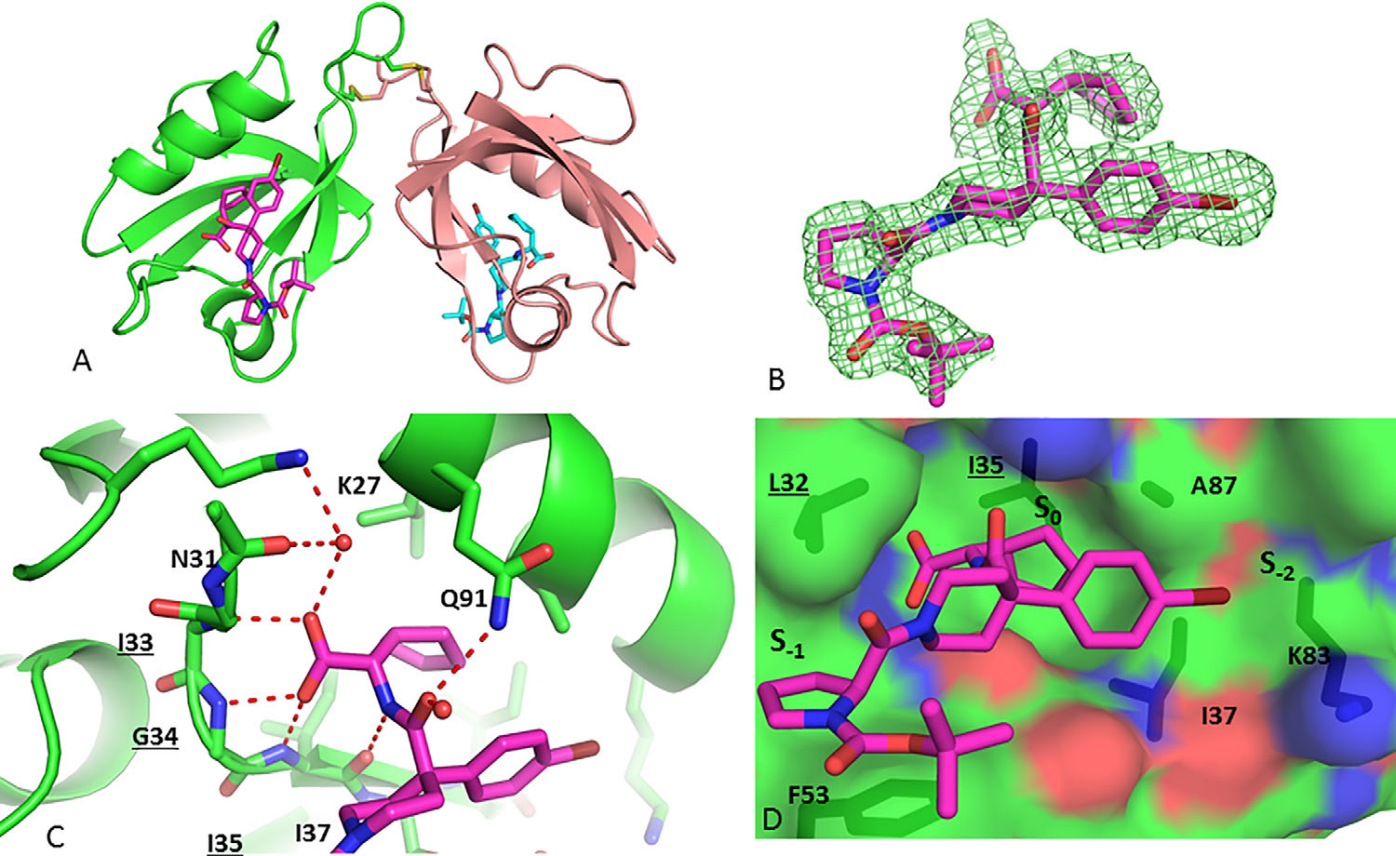
(A) Overall Structure of PICK1 PDZ‐QS with BIO124 bound in the peptide binding pocket. (B) Fo‐Fc electron density seen for BIO124. Hydrogen Bond network (C) and Hydrophobic Interactions (D) observed between BIO124 and the peptide binding Pocket of PICK1. Residues of the Carboxylate binding loop are underlined. An interactive view is available in the electronic version of the article

The crystal structure of PICK1‐PDZ‐QS/BIO124 was solved to 1.75 Å and is similar to the previously solved PICK1‐QSAV dimer structure except for the peptide binding region. The electron density begins at Val19 and ends at Leu104, and stops just prior to the C‐terminal extension. The structure has one dimer molecule per asymmetric unit as described in the QSAV‐structure [Fig. 4(A)]. The *F*_o_ – *F*_c_ electron density maps clearly showed that BIO124 was present in molecule A and B [Fig. 4(B)], and thus a “lock and chop” method through crosslinking and proteolysis successfully produced a compound‐bound PDZ domain co‐crystal structure for PICK1.

### BIO124 binds in an extended conformation at the surface of the peptide binding pocket

As expected, BIO124 is anchored into the peptide binding pocket through several hydrogen bonds [Fig. 4(C)]. Its carboxylic acid is involved in an intricate network of hydrogen bonds to the backbone of three amino acids (Ile33, Gly34, and Ile35) of the carboxylate binding loop. There is also a water‐mediated interaction to the backbone carbonyl of Asn31 and the side chain amine group of Lys27. In addition, the peptide linker of BIO124 hydrogen bonds to the top and bottom of the pocket through backbone interactions with the amide of Gln91 and carbonyl of Ile35. The cyclopropyl group makes lipophilic interactions with Ile37, Ala87, and Ile90 within the S_0_ sub‐pocket [Fig. 4(D)]. The piperidine core occupies the −1 position and provides two vectors for additional lipophilic interactions. The pyrolidine is positioned to stack between Leu32 and Phe53 in the S_–1_ sub‐pocket and the bromophenyl moiety travels from the −2 position toward the aliphatic segment of K83 in S_–2_ sub‐pocket. The piperidine group of Bio124 is important to make multiple contacts with PICK1, the lowest energy conformer places the bromophenyl in the equatorial position which forces the amide into the axial position and allows the piperidine to make hydrophobic contacts underneath the carboxylate binding loop as it reaches into the −1 position sub‐pocket. This extended binding mode shows that additional interactions can be made outside the peptide binding pocket which could be optimized to improve potency and/or selectivity for this class of inhibitors.

**Figure 5:**
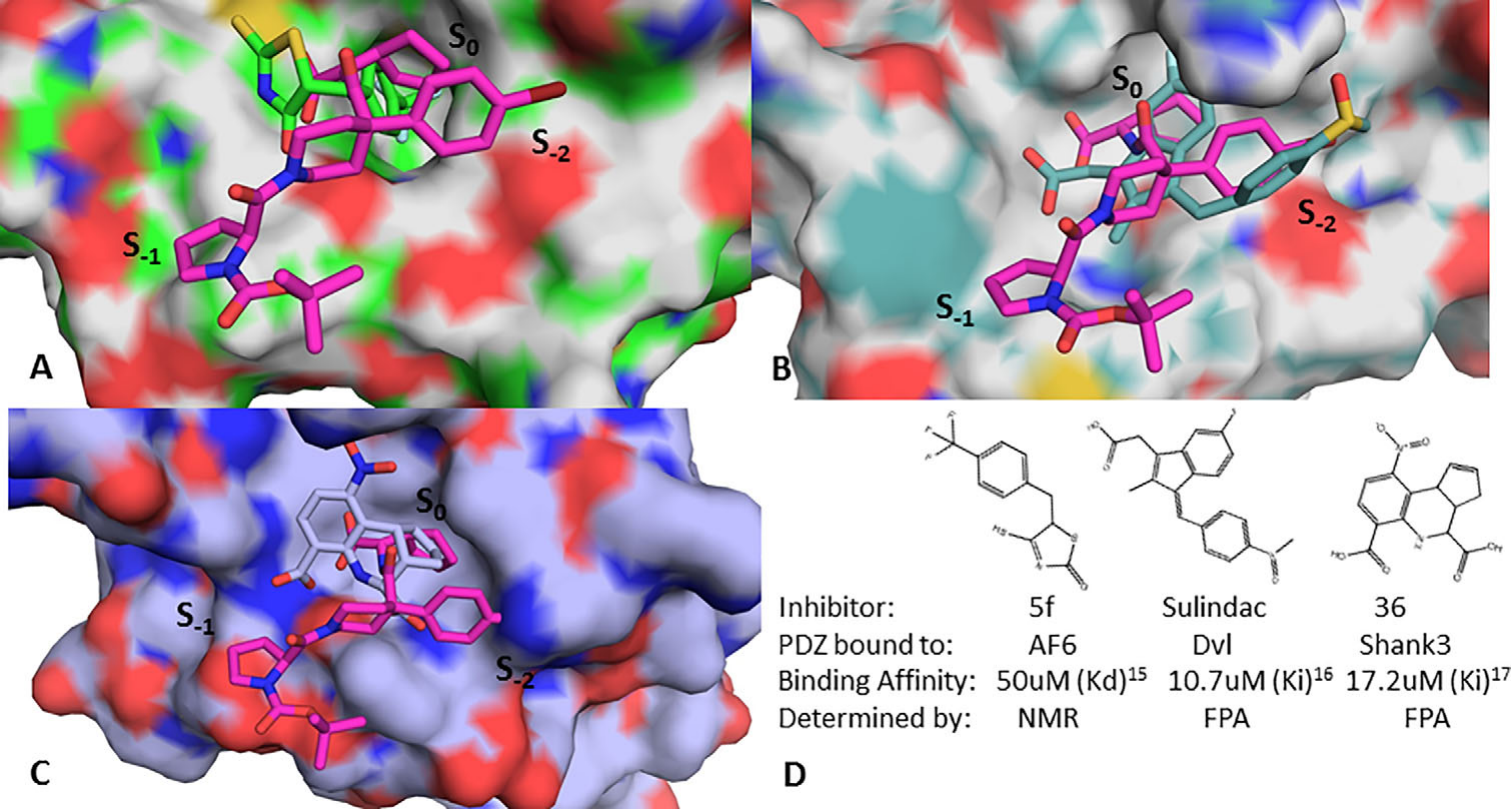
Overlay of BIO124 with the costructures of (A) AF6 5f complex, (B) Dvl Sulindac complex and (C) Shank compound 36 complex. (D) Binding affinities and assay method for PDZ domain inhibitor costructures.

On comparing the binding mode of BIO124 to PICK1 with the published small molecule inhibitors bound to their PDZ structures including AF6 [Fig. 5(A); PDB ID: 2EXG], Dishevelled (Dvl) [Fig. 5(B); PDB ID: 2KAW], and Shank3 [Fig. 5(C); PDB ID: 3O5N], we find key interactions missing between the respective inhibitors and their PDZ domain that could explain their relatively weak binding affinities [Fig. 5(D)].^13^, ^21^, ^22^ In all three cases, the inhibitors form hydrogen bond interactions with the carboxylate binding motif and fill the S_0_ sub‐pocket making the lipophilic interactions which anchor them into the peptide binding pocket. Yet, all three compounds from these known small molecule/PDZ structures exhibit modest inhibition potencies at 50 μM, 10.7 μM, 17.2 μM for AF6, Dvl, and Shank3, respectively [Fig. 5(D)].^13^, ^21^, ^22^ Of the three, Sulindac bound to Dvl is the only inhibitor that binds into the S_–2_ sub‐pocket, by making a hydrogen bond to Arg332 of its αB helix. None of the molecules extend very far into their respective S_–1_ sub‐pocket. In contrast, BIO124 extends into the S_–1_ sub‐pocket and uses its pyrolidine moiety to form lipophilic interactions with Leu32 of the carboxylate binding loop and Phe53. This leads us to suggest that the binding affinities of these ligands for their respective PDZ domains could be improved by making additional interaction at the surface of the peptide binding pocket either by extending into the S_–1_ sub‐pocket or toward the αB1 position as seen for BIO124.

### Future direction in generating PDZ small molecule co‐crystal structures using the lock and chop method

Elkins et al. demonstrated that the addition of a self‐binding C‐terminal PDZ binding motif to PDZ domains can enhance solubility and promote crystallization.^18^ This methodology has proven useful in generating novel PDZ domain crystal structures and mapping interactions with PDZ binding motifs. In this study, we used this method to generate a PICK1 PDZ domain structure in complex with the class I PDZ binding motif peptide from PKCα. In addition, by using a C‐terminal proteolysis step to liberate the C‐terminal QSAV extension from the peptide binding site, we achieved the co‐crystallization of the PICK1 PDZ domain with BIO124. The crosslinking of the dimer through cysteine oxidation was critical for locking and maintaining the dimeric state of the protein for crystallization. We hypothesize that this method could be used to enable other PDZ domain small molecule inhibitor co‐crystal structures. Here, after a self‐binding C‐terminal PDZ binding motif extension has been identified for producing a crystal structure, site directed mutagenesis could be used to introduce interactions to lock the PDZ domain into its crystallization competent form. Once locked, small molecule inhibitors targeting the peptide binding site could be introduced to liberate the C‐terminal peptide extension for proteolytic digestion to produce a PDZ small molecule inhibitor complex to enter crystallization trials.

In summary, we report a novel method for producing co‐crystal structures of the PDZ domain of PICK1 with a small molecule inhibitor. Our PICK1 PDZ BIO124 co‐crystal structure demonstrates a new extended compound binding mode in which lipophilic interactions are made with Lys83 at the αB1 position and in the −1 position sub‐pocket spanning the peptide binding site of PICK1.

## Materials and Methods

### Cloning, protein expression, and purification

PICK1 PDZ–QSAV was constructed as follows: cDNA for human PICK1 encoding residues 1 to 105 were cloned into a pET15b vector in frame with a TEV cleavable N‐terminal HIS tag and the C‐terminal fused QSAV tail (PICK1 PDZ‐QSAV). BL21 (DE3) Escherichia coli cells transformed with PICK1 PDZ‐QSAV were grown at 37°C in LB media supplemented with ampicillin to an OD600 of 1, at which point the temperature was reduced to 18°C and protein expression was induced with 1 mM IPTG. After 16 hours, the cells were harvested and resuspended in lysis buffer (25 mM TRIS‐HCl pH 8.0, 250 mM NaCl, 10% (v/v) glycerol, 2.5 mM β‐mercaptoethanol (β‐ME), 20 mM imidazole, and Roche EDTA‐free protease inhibitor cocktail) and subjected to two passes through a microfluidizer. The lysate was clarified by centrifugation at 20,000*g* for 1 hour at 4°C, and PICK1 PDZ‐QSAV was captured by batch binding to nickel resin for 16 hours at 4°C. The nickel resin was washed with buffer A (25 mM TRIS‐HCl pH 8.0, 250 mM NaCl, 10% (v/v) glycerol, 2.5 mM β‐ME, and 20 mM imidazole) and then loaded into an XK16/20 column and washed to baseline on an AKTA purifier. PICK1 PDZ‐QSAV was eluted from the nickel column using buffer A supplemented to 250 mM imidazole and analyzed by SDS‐PAGE. The PICK1 PDZ‐QSAV domain was purified further using a Superdex 75 gel filtration column equilibrated in buffer B (25 mM TRIS‐HCl pH 8.0, 250 mM NaCl, 5% (v/v) glycerol, and 2 mM DTT). PICK1 PDZ‐QSAV eluted as a dimer and was approximately 95% pure based on SDS‐PAGE. The PICK1 PDZ‐QSAV was flash frozen and stored at −80°C.

### Discovery of BIO124

HTS screening was done using a Fluorescent Polarization (FP) assay with the PICK PDZ‐BAR construct as described in Alfonso et al.^5^ After several rounds of hit optimization, compound BIO124 was identified with an IC50 of 513 nM in the FP assay [Fig. 1(B)].

### PICK1 PDZ‐QSAV inter‐disulfide bridge formation

PICK1 PDZ‐QSAV concentrated to 3 mg/mL was treated with 2.5 mM H_2_O_2_ and incubated for 16 hours at 4°C to form intermolecular disulfide bridges which was expected to lock the protein into a dimeric form. Dimer formation was confirmed by nonreducing SDS PAGE. Disulfide‐linked dimeric PICK1 PDZ‐QSAV was separated from higher order PICK1 PDZ‐QSAV species using a Superdex 75 column equilibrated in buffer C: 25 mM TRIS‐HCl pH 8.0, 250 mM NaCl, and 5% (v/v) glycerol and concentrated to 3 mg/mL.

### PICK1 PDZ‐QSAV differential scanning fluorimetry

The thermal denaturation (Tm) curves for the unlocked and locked dimer of PICK1 PDZ‐QSAV were determined using a QuantStudio 12K Flex QPCR instrument. The assay was carried out in 25 mM TRIS‐HCl pH 8.0, 250 mM NaCl, 5% (v/v) glycerol, and 1x Protein Thermal Shift dye (Life Technologies) with the PICK1 PDZ‐QSAV proteins at 1 µM. The proteins were denatured from 20 to 85°C with a 1°C/minute gradient and fluorescence measured every 0.5 seconds.

### Crystallization of dimeric PICK1 PDZ‐QSAV in the presence of BIO124

Dimeric PICK1 PDZ‐QSAV at 3 mg/mL (220 μM) was incubated with 4.5 molar equivalents of BIO124 for 2 hours on ice and then co‐concentrated to 10 mg/mL for crystallization. Crystals of dimeric PICK1 PDZ‐QSAV grew from 0.1 M BisTRIS pH 5.5 and 25% PEG3350 (w/v) at 4°C in 2 days. Crystals were cryoprotected in the mother liquor supplemented with 20% (v/v) glycerol prior to being frozen in liquid nitrogen.

### Data collection and structure determination of the PICK1 PDZ QSAV

X‐ray diffraction data for crystals of dimeric PICK1 PDZ‐QSAV with BIO124 was measured using a Rigaku FRE (Rigaku, The Woodlands, TX) and was processed with HKL2000.^12^ The crystals belonged to a P3_2_ space group with one disulfide‐linked PICK1 PDZ‐QSAV dimer per asymmetric unit. The structure was solved with MOLREP using a PICK1 PDZ domain structure (PDB code 2GZV) in which the last 4 residues were removed.^14^, ^18^ The initial model was subjected to several rounds of refinement and model building using Refmac5 and Coot.^15^, ^16^ The final model had an *R*_work_ of 19.4% and *R*_free_ of 22.0% to 2.44 Å with good geometry (Table 1). The structure has been deposited with the PBDID: 6BJN.

**Table 1:** Collection and Refinement Statistics for PICK1‐PDZ‐QSAV and PICK1‐PDZ‐BIO124 Crystal Structures

Data collection	PICK1‐PDZ‐QSAV PDB:6BJN	PICK1‐PDZ‐BIO124 PDB:6BJO
Space group	*P3_2_*	*P3_2_*
Cell dimensions		
Unit cell length (Å)	54.47 × 54.47 × 78.12	54.01 × 54.01 × 77.87
Unit cell angles (°)	90 × 90 × 120	90 × 90 × 120
Wavelength (Å)	1.54	0.97
Resolution (Å)	50–2.44	50–1.75
*R*_sym_ a	0.19 (0.74)	0.04 (0.53)
I/σ	7.1 (1.5)	13.9 (1.29)
Multiplicity	4.8 (4.5)	2.9 (1.8)
Total no. reflections/no. unique reflections	46,584/9,544	73,120/25,511
Mean I/σ	7.1 (1.5)	13.9 (1.29)
Completeness (%)	99.6 (99.6)	98.3 (99)
Rwork^b^ (Rfree)	19.4/22.0	13.9/15.7
CC_1/2_	0.86(0.69)	0.9 (0.51)
No. molecules per asymmetric unit	2	2
R.m.s.d. bond distance (Å)	0.016	0.038
R.m.s.d bond angle (deg)	1.92	3.8
Total no. of non‐H atoms in ASU	1,385	1,477
No. of solvent molecules	31	164
Avg. protein B‐value (Å^2^)	39.3	21.8
Avg. solvent B‐value (Å^2^)	46.4	44.38
Ramachandran plot		
Preferred	95.6	93.96
Generous	4.4	6.04
Disallowed	0.0	0.0

*The value in parentheses is for the highest resolution bin (approximate interval, 0.1 A).

a Rsym = ƒ°| ƒ§hkl.≤ ƒ§hkl ≥|/ƒ° ƒ§hkl.

b Rwork = ƒ°ƒ§hkl| |Fo . |Fc| |/ƒ°ƒ§hkl|Fo| for all data except 5% which is used for the Rfree calculation.

### PICK1 PDZ‐QSAV NMR chemical shifts

To produce methylated PICK1 PDZ material for NMR, dimeric PICK1 PDZ‐QSAV surface lysines were methylated with 19C‐labeled formaldehyde as described in Macnaughtan et al.^17^ 0.8 mM protein in 50 mM sodium phosphate pH 6.0 was incubated with an equivalent of 19C formaldehyde (Sigma‐Aldrich) for 1 hour at room temperature before addition of about 15 equivalents of sodium cyanoborohydride (Fluka). Reactions were left to proceed for 12 hours before extensive buffer exchange in the final NMR buffer; 40 mM sodium phosphate pH 6.8, 150 mM NaCl, 0.2 mM EDTA, 2% (v/v) DMSO in D_2_O. The wild type GluA2 peptide (VYGIESVKI, New England Peptide) or BIO124 in 100% DMSO were added (4% final DMSO concertation) to a final concentration of 500μM or 800 μM dimeric PICK1 PDZ‐QSAV in NMR buffer, respectively. ^1^H‐^13^C‐HSQC experiments were acquired at 298 K on a 600 MHz Bruker NMR spectrometer equipped with a 1.7 mm TCI cryoprobe and processed using TopSpin 3.2 (Bruker).

### Proteolytic treatment of dimeric PICK1 PDZ‐QSAV using carboxypeptidase a in the presence of BIO124

Dimeric PICK1 PDZ‐QSAV at 3mg/mL (220 µM) was incubated with 4.5 molar equivalents of BIO124 for 2 hours on ice. Bovine Carboxypeptidase A (bCPA) (Sigma Aldrich, St. Louis, MO) was diluted in 25 mM TRIS‐HCl pH8.0, 250 mM NaCl, 5% (v/v) glycerol, and 2.5 mM ZnCl_2_ and added to the dimeric PICK1 PDZ‐QSAV/BIO124 complex at a 1:15 molar ratio and allowed to incubate for 16 hours at 4°C to proteolyze the QSAV tail. The reaction was quenched with the addition of 5 mM EDTA pH 8.0.

### Mass spectrometry analysis of the PICK1 PDZ‐QS BIO124 complex

bCPA‐treated PICK1 PDZ‐QSAV domain BIO124 complex was reduced with 50 mM dithiothreitol in Tris‐buffered saline, pH 8.0, containing 4 M urea and 5 mM EDTA. The bCPA‐ treated PICK1‐PDZ BIO124 complex was then analyzed on an LC‐MS system comprised of a UPLC (ACQUITY, Waters Corp.), a TUV dual‐wavelength UV detector (Waters Corp.), and a ZQ mass spectrometer (Waters Corp.). A Vydac C4 cartridge was used for desalting. Molecular masses for the bCPA‐treated PICK1‐PDZ BIO124 complex were obtained by deconvolution of raw mass spectra using the MaxEnt 1 program embedded in MaxLynx 4.1 software (Waters Corp.).

### Dimeric bCPA‐treated PICK1 PDZ‐QS BIO124 cocrystallization

bCPA‐treated PICK1 PDZ‐QS BIO124 complex was concentrated to 10 mg/mL. PICK1 PDZ BIO124 crystals were grown by seeding the drop with PICK1 PDZ‐QSAV crystals in 0.1 M BisTRIS pH 5.5 and 25% PEG3350 (w/v) at 4°C. Crystals of the PICK1 PDZ BIO124 complex were cryoprotected for data collection by transferring them to 0.1 M BisTRIS pH 5.5 and 25% PEG3350 (w/v) and 20% glycerol prior to being frozen in liquid nitrogen.

### Data collection and structure determination of bCPA‐treated PICK1 PDZ‐QS BIO124 complex

X‐ray diffraction data for crystals of dimeric bCPA‐treated PICK1 PDZ‐QS BIO124 was measured at beam line 31ID at the Argonne Photon Source and was processed with HKL2000.^12^ The crystals belonged to a P3_2_ space group with one disulfide‐bridged PICK1 PDZ BIO124 dimer per asymmetric unit. The structure was solved with MOLREP using the PICK1 PDZ‐QSAV structure with the AV removed as the search model.^14^ The structure went through multiple rounds of refinement and model building using Refmac5 and Coot which led to a model with *R*_work_ of 13.9% and *R*_free_ of 15.7% to 1.75 Å with good geometry (Table 1).^15^, ^16^ The structure has been deposited with the PBDID: 6BJO.

## Disclosure

The authors declare that they have no conflicts of interest with the contents of this article.

## Supporting information

Supporting Information Figure 1

Supporting Information Figure 2

Supporting Information Figure 3
