# Supplementary figures and images for: Lock and chop: A novel method for the generation of a PICK1 PDZ domain and piperidine‐based inhibitor co‐crystal structure
Source: Protein Sci. 2018 Jan 30;27(3):672–80. doi: 10.1002/pro.3361 (PMC5818740; doi:10.1002/pro.3361)

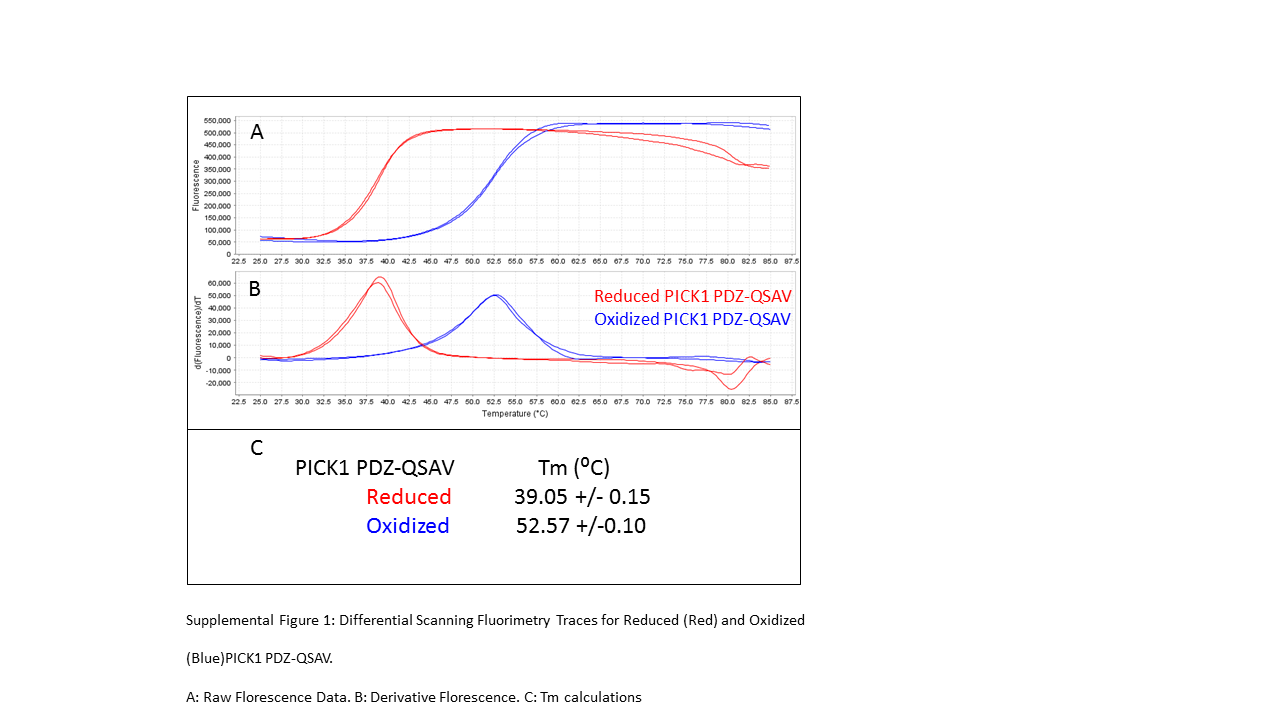

Supplement: Supplementary file 1 — Supporting Information Figure 1 [file PRO-27-672-s001.TIF]

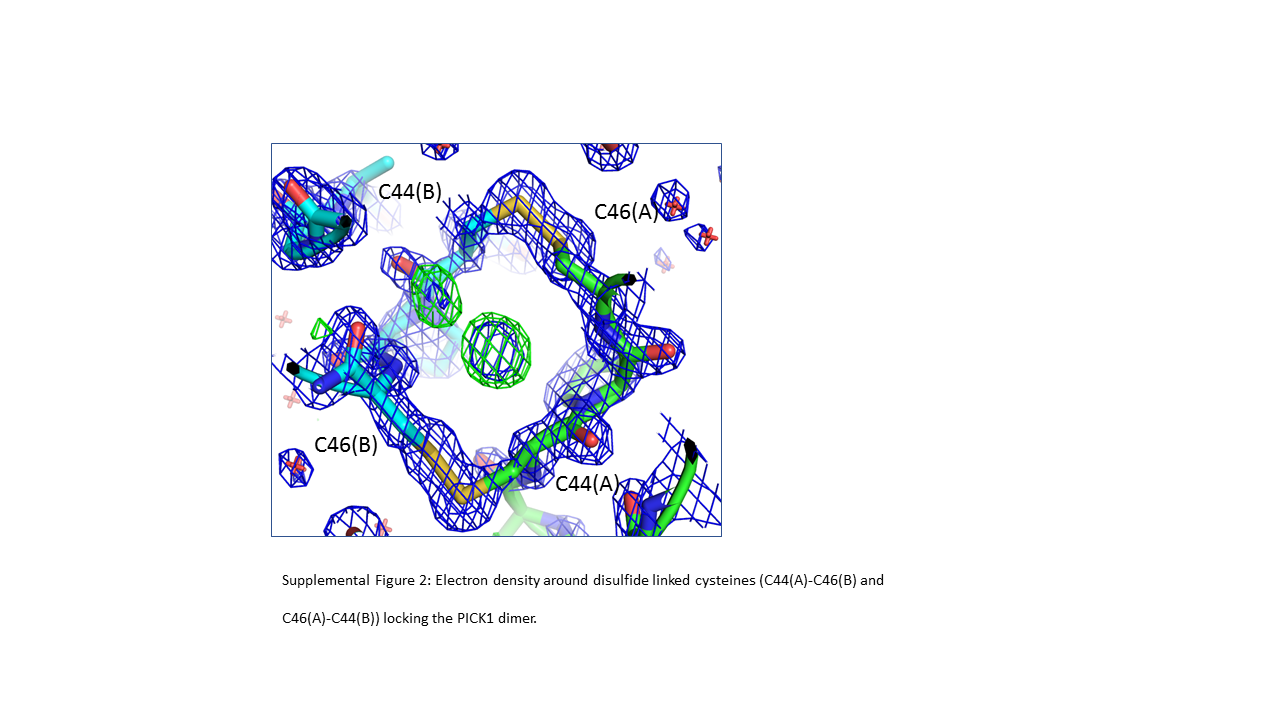

Supplement: Supplementary file 2 — Supporting Information Figure 2 [file PRO-27-672-s002.TIF]

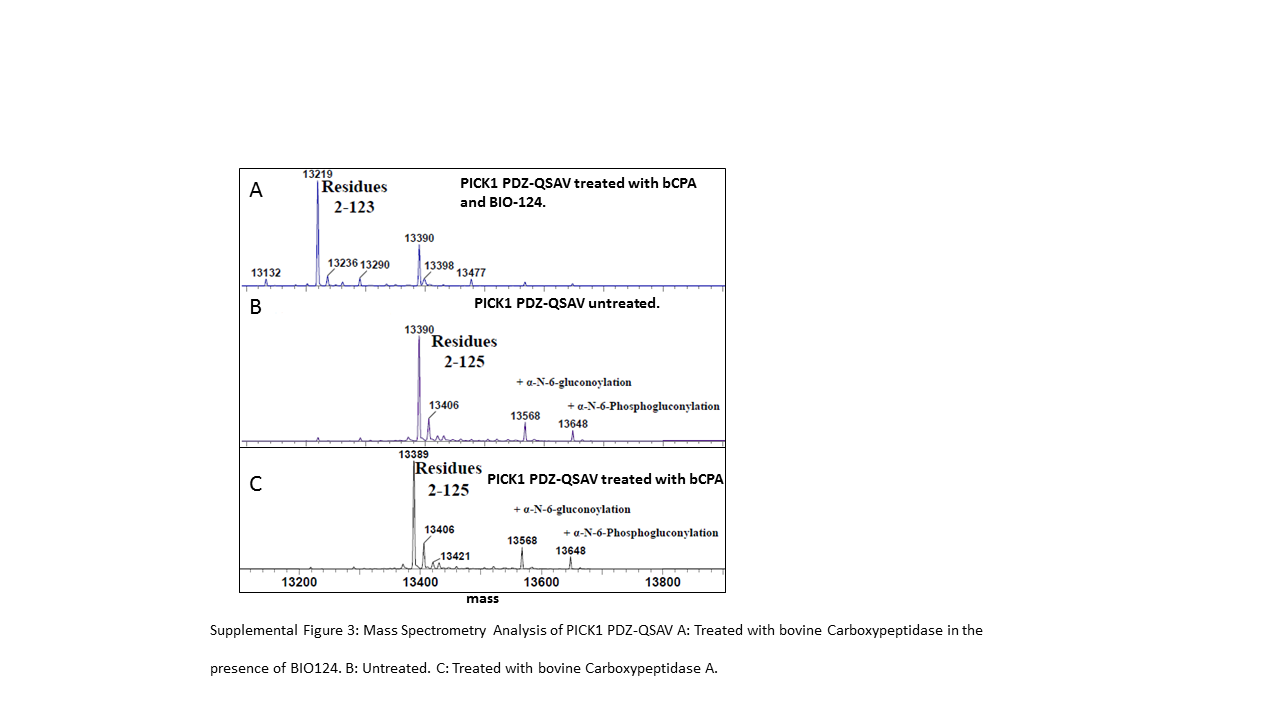

Supplement: Supplementary file 3 — Supporting Information Figure 3 [file PRO-27-672-s003.TIF]
